# The non-human animal reading the mind in the eyes test (NARMET): A new measure for the assessment of social cognition

**DOI:** 10.3389/fpsyt.2023.1129252

**Published:** 2023-03-20

**Authors:** Clare M. Eddy

**Affiliations:** ^1^BSMHFT National Centre for Mental Health, Birmingham, United Kingdom; ^2^College of Medical and Dental Sciences, University of Birmingham, Birmingham, United Kingdom

**Keywords:** alexithymia, animal images, assessment, emotion, empathy, measures, social cognition

## Abstract

The Reading the Mind in the Eyes test (RMET) is a widely applied test of social cognition, based on mental state judgments in response to photographs of human eyes, which can elicit impairment in patients with numerous psychiatric and neurological disorders. However, interpretation of task performance is limited without the use of appropriate control tasks. In addition to a matched task requiring age judgments of the RMET stimuli, it was recently shown that a mental state judgment task of comparable difficulty, could be developed using photographs of domestic cat eyes. The current study aimed to further develop a Non-human Animal RMET (NARMET) by testing additional stimuli in the form of photographs of domestic dog eyes. A variety of additional tasks were used alongside the eyes test stimuli in a large sample of healthy young adults, to explore how alexithymia, schizotypal features, and autistic tendencies may differentially influence mental state attribution in response to cat, dog, and human eyes test stimuli. The resulting NARMET features both cat and dog trials, depicting a similar range of complex mental states to the human RMET. It shows favorable psychometric properties as well as being well matched to the RMET in terms of linguistic variables, length and difficulty. However, reading measures predicted performance on the RMET, but not on the NARMET. Although further testing is required in samples with a higher proportion of males, future application of the NARMET in neuropsychiatric populations exhibiting cognitive and behavioral difficulties could offer enhanced assessment of social cognitive skills.

## Introduction

The Reading the Mind in the Eyes test (RMET) is a widely applied test of social cognition, involving selection of complex mental states to match photographs of the human eye region (1). Use of this task has revealed impairments in numerous clinical populations including those with a diagnosis of schizophrenia (2, 3), Huntington’s disease (4), and brain injury (5). Performance on the RMET is thought to be related to empathy (1, 6) and alexithymia (7), in addition to schizotypal personality (8, 9), and autistic spectrum features (6, 10, 11). However, the exact skills underlying performance on the task are still debated. Some scholars describe the task as involving emotion recognition (12) while others believe it measures abstract mentalizing about mental states (1). To gain insight into the reasons for any performance deficits, it is important to consider the use of matched control tasks, such as the age judgment eyes task (9, 13, 14).

In one recent study, it was shown that a counterpart measure of comparable difficulty to the human RMET could be developed using photographs of domestic cat eyes (9). Cats were selected because they familiar to humans, exhibit a similar layout of facial features, and an abundance of images were available online. It is known that humans attribute human-like personality traits such as curious, playful, and serious to their pet cat (15). There was a high level of consensus in relation to the attribution of secondary emotions on the cat eyes task (9), which followed the format of the human RMET, and comprised 18 photographs of the cat eye region surrounded by the same forced-choice complex mental state terms featured in the original task (e.g., preoccupied, decisive, and tentative). Performance on the RMET and cat eyes task was highly correlated, but while RMET accuracy was predicted by working memory, schizotypal personality and measures of empathy toward humans, cat eyes test responses were not, suggesting that stimuli featuring human versus non-human animals may be differentially impacted by psychopathology. The current study aimed to further develop a non-human animal version of the RMET.

It was previously found that higher accuracy on both the RMET and cat eyes test was positively associated with ratings of liking dogs on a simple animal preference questionnaire (9). Given this finding and the proposed existence of “cat” people and “dog” people (16), it was decided to expand the stimulus set to also include dog eyes, as in addition to adding variety, dogs should be familiar to test subjects, and work in a similar way to cats. As well as having have many of the same muscles that produce facial expressions in humans (17), dogs are adept at interacting with humans, and react to human affective states (18, 19). It has even been suggested that dogs seem to use facial movements (e.g., brow lift, jaw drop, and tongue show) specifically when their owner is able to view these cues (20). Personality judgments about dogs and humans have been shown to reach similar levels of agreement and consistency (21), projection of self-views onto dogs appears no more likely than onto other humans (22), and some traits attributed to dogs appear to reflect a consistent personality or temperament (23). While it is important to note the potential differences between personality traits, emotions and non-emotional mental states, humans generally agree that dogs can experience emotions (24), including secondary emotions e.g., jealousy and guilt (25). Basic emotional expressions in dogs are reported by 65–100% of dog owners (25, 26), and are reported to be slightly more frequent in dogs than cats (25). It was therefore expected that a similar degree of consensus would be found for newly created dog eyes test stimuli as was found for the cat eyes test (9).

A variety of additional tasks were used alongside the eyes test stimuli to explore whether psychological factors may differentially influence performance across the human and non-human animal versions of the tasks. Because vocabulary and verbal fluency can be related to RMET performance (27, 28), measures of reading were included. Previously identified associations with empathy were further explored by including measures associated with emotion processing (alexithymia; emotion contagion; mood). Measures of anthropomorphism, and motivation associated with understanding mental states were also included because participants were asked to attribute mental states to non-human animals. Scales for autistic features, schizotypal personality characteristics, and social anhedonia were included, to test for differential relationships with social cognition in relation to human versus non-human stimuli. Based on previous findings (9), it was predicted that responses to all types of eyes stimuli would correlate with aspects of empathy, but that performance on the human and animal stimuli would be differentially predicted by measures associated with psychopathology, with more relationships of this nature being expected for the human RMET.

## Materials and methods

### Participants

After ethical permission was granted for the study by University of Birmingham, 210 undergraduate students without any current psychiatric or neurological diagnoses, or cat/dog phobia, volunteered to participate for course credit. Five had missing data and 1 demonstrated below chance performance on the eyes tests. A further three participants had incomplete data on a few questionnaires but were included after imputation of missing values based on group mean (29). The final sample consisted of 204 participants (189 females; 15 males), of mean age 19.81 ± 0.93 years, median = 19.7; range = 18.26–23.95 (age was not normally distributed, 95% CI: 19.68, 19.94).

### Procedure

Participants completed the tasks individually in a lab at the University. They provided demographical information (gender, date of birth, year of study), and completed the Hospital Anxiety, and Depression Scale (HADS), Test of Irregular Word Reading Efficiency (TIWRE), and Test of Word Reading Efficiency (TOWRE). Participants then completed the three computerized eyes tasks, which were presented using Presentation (Neurobehavioral Systems) software, after being provided with Baron-Cohen et al.’s glossary of mental state terms. The order of presentation of each stimuli set (human RMET, cats, and dogs) was counterbalanced across participants. These were followed by computerized questionnaires: Interpersonal Reactivity Index (IRI), Toronto Alexithymia Scale (TAS-20), Revised Social Anhedonia Scale (rSAS), Autism Spectrum Quotient (AQ50), Emotional Contagion Scale (ECS), Individual Differences in Anthropomorphism Questionnaire (IDAQ), Mind Reading Motivation scale (MRM), and Oxford-Liverpool Inventory of Feelings and Experiences (O-Life).

### Measures

#### TIWRE and TOWRE

Participants read out loud 108 regular words and then 39 irregular words (30, 31) with no time limit. TOWRE includes part A (sight reading efficiency) and part B (phonemic decoding efficiency). Errors and time taken were recorded.

#### IRI

The IRI (32, 33) contains 4 subscales each with 7 items. Perspective taking (PT) assesses the tendency to adopt other people’s points of view, and empathic concern (EC), addresses feelings of warmth, and consideration toward others. High scores for personal distress (PD) indicate more feelings of negative emotion when around other people in distress and the fantasy subscale measures the propensity to imagine and relate to characters in books and films. Participants respond on a 5 point Likert scale based on how well each item describes them. Some items are reverse scored, and total score ranges from 0 to 112.

#### TAS-20

The TAS assesses alexithymia and has good reliability and construct validity (34–36). There are three subscales: difficulty identifying feelings (DIF e.g., “I have feelings that I can’t quite identify”); difficulty describing feelings (DDF e.g., “It is difficult for me to find the right word for my feelings”), and externally oriented thinking (EOT e.g., “I prefer to just let things happen rather than to understand why they turned out that way”). Some items are reverse scored. Scores can range from 20 to 100, with a cut of at 61 being proposed to identify alexithymic individuals.

#### rSAS

The revised SAS (37) contains 40 items and assesses social withdrawal and lack of pleasure from social relationships e.g., “A car ride is much more enjoyable if someone is with me”; “Having close friends is not as important as some people say.” A cut-off of 12 or over has been proposed (38) with higher scores indicating more social anhedonia. Some items are reverse scored.

#### IDAQ

This self-report measure (39) contains 30 items in total and is used to assess anthropomorphic tendencies whereby participants are asked to rate how much they believe a non-human entity (e.g., wind; robot etc.) possesses human characteristics on a scale of 0 (not at all) to 10 (very much). For example, items include “To what extent does the average computer have a mind of its own?” and “To what extent does the average reptile have consciousness?” The two subscales (each 15 items) measure anthropomorphic (mental state related) and non-anthropomorphic attribution (attributions related to clearly observable or functional aspects of a stimulus).

#### ECS

This scale (40, 41) contains 15 items and is used to assess individual differences in susceptibility to feeling the emotions exhibited by other people, including love, happiness, fear, anger, and sadness.

#### HADS

This self-report measure (42) was developed to assess anxiety (7 items) and depression (7 items) that a person is experiencing.

#### MRM

This scale assesses tendencies toward thinking about mental states rather than ability (43). The developers showed it can be associated with teamwork, is stable over time, and goes beyond trait empathy.

#### O-Life

This scale (44) assesses unusual experiences, cognitive disorganization, introvertive anhedonia, and impulsive non-conformity. Psychometric testing indicated high internal consistency (45) and test-retest reliability (46).

#### AQ

The AQ (47) consists of 50 statements, each of which is in a forced choice format with 4 ratings ranging from “definitely agree” to “definitely disagree.” Neurotypical individuals would agree with half of the statements and disagree with half. The statements are related to five different domains relevant to autistic traits: social skills; communication skills; imagination; attention to detail; and attention switching/tolerance of change. Statements answered in a fashion associated with autistic tendencies score a point. A score of 32 or more is thought to be indicative of high autistic traits.

#### Eyes task stimuli

The RMET (1, human eyes) contains 36 test trials plus one practice trial. Stimuli consist of photographs of the human eye region, surrounded by four mental state terms (e.g., terrified, upset, arrogant, and annoyed). Instructions require the participant to consider these terms and select the word they think is most appropriate to describe what the person in the photograph is thinking or feeling. Correct answers provided by Baron-Cohen et al. (47) were determined based on consensus across expert judges. Evidence of task validity comes from correlations of other measures of mental state, reasoning and recognition such as the faux pas test (48), and the ability of this task to identify individuals with known deficits in social cognition (49). The RMET has good test-retest stability as shown over 1 year in a non-clinical sample (50).

The development of the cats eyes test was described previously (9). Trials were designed to match corresponding RMET trials, and the best subset of trials (*n* = 18) was selected to match to the RMET for difficulty, and to achieve sufficient reliability and internal consistency. Correct answers were based on consensus (majority response for each item in the current study were no different to our previous study). The dog eyes test stimuli were developed for the current study following the same process as for the cats eyes stimuli i.e., 36 images (plus 1 for practice) without re-use restrictions were selected from the internet, based on visual similarity to the human stimuli (e.g., eye shape, direction of gaze), with a view to matching each individual RMET trial. Each eyes test commenced with onscreen instructions to pick “the word that best describes what the person/cat/dog in the image is thinking or feeling.” Images were approximately 28 cm × 9 cm high (24″ monitor; resolution 1,024 × 768), with response options in Arial 22 point (approximately 1 cm high) outside the corners of the image, mapped to the numeric keypad which was used to respond (1, 3, 7, 9). The first trial began after the participant pressed the space bar. There was no time limit, and a valid button response initiated the next trial.

### Analysis

Cat stimuli were validated using two separate samples in a previous study (9). The current paper presents additional analysis involving the cats stimuli (18 trials), and reports data on the dog eyes test stimuli for the first time. As with the cat eyes test, accuracy was determined based on consensus (initial sample majority responses) for the dog stimuli. 475 trials were removed (1.1% of 44,064 trials in total: 36 × 6 × 204) of data due to responses less than 200 ms, or more than mean + 3 times SD per condition. After determining the correct answers and the best subset of 18 dogs eyes test trials to match the RMET for accuracy, this accuracy match was tested again in a second sample (*n* = 228), as was done previously (9) for the cat stimuli. Further comparisons of accuracy and reaction times were made across the three sets of eyes stimuli. Reliability was also compared for human, cat, and dog stimuli, before investigating relationships between eyes test performance, and other measures. Normality was tested using Shapiro-Wilk, and a Box-Cox transformation was performed (λ = 2), consistent with our previous study that developed the cat stimuli (9).

## Results

Accuracy scores (%) and mean reaction times (RT; seconds) for the human (72.61 ± 0.12; RT 3.56 ± 1.07), cat (71.81 ± 0.14; RT 3.29 ± 1.07), and dog (72.53 ± 0.15; RT 3.36 ± 1.02) stimuli were well matched. Mean accuracy for all cats plus all dogs was 72.17 ± 0.15, RT 3.33 ± 1.05 s. Descriptive statistics for the full set of measures can be found in Supplementary Table 1. Few participants within the sample exhibited scores above cut-offs for scales such as the AQ50 (*n* = 4) and TAS-20 (*n* = 20), although more participants scored above the cut-off for the rSAS (*n* = 44).

### Eyes stimuli performance comparisons

**FIGURE 1:**
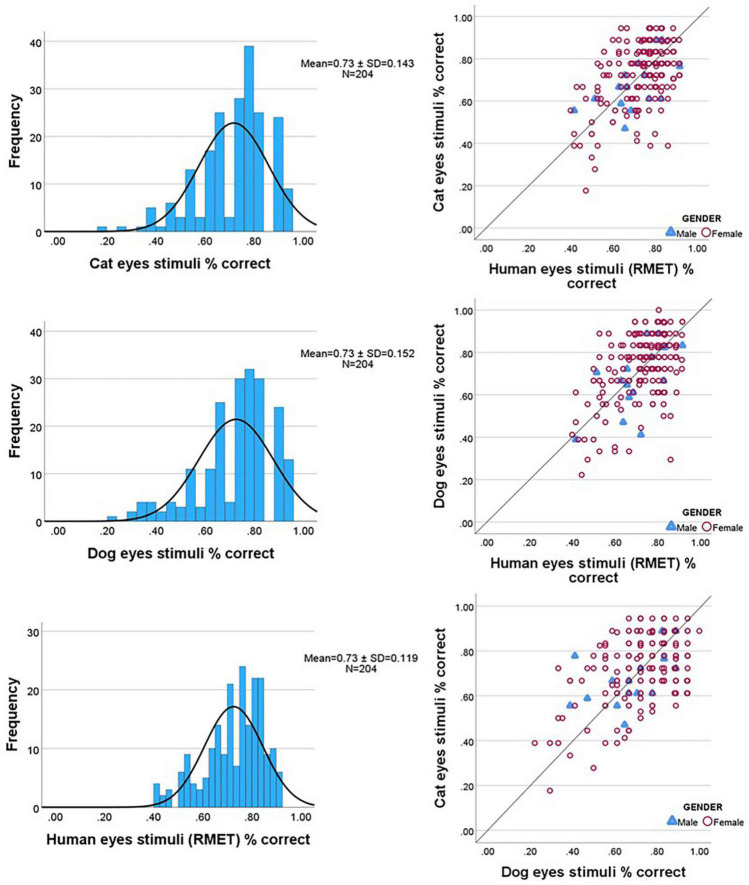
Frequency distributions and correlation plots for eyes task stimuli scores.

Correlations between the eyes tests were strong and positive, suggestive of convergent validity: dogs and cats: *r* = 0.423, *p* < 0.0001; cats and human RMET: *r* = 0.483, *p* < 0.0001; dogs and human RMET: *r* = 0.468, *p* < 0.0001. Pairwise *t*-tests indicated no significant differences between any two stimulus sets [dogs and cats: *t*(203) = −0.873, *p* = 0.384; cats and human RMET: *t*(203)-0.465, *p* = 0.642; dogs and human RMET: *t*(203) = 0.508, *p* = 0.612]. Good performers on one type of eyes stimuli tended to score highly on the other types. Eyes stimuli score distributions and correlation plots are shown in Figure 1.

Participants took slightly longer to respond to human RMET trials in comparison to cat [*t*(203) = −5.334, *p* < 0.001] and dog trials [*t*(203) = −4.214, *p* < 0.001]. There was no difference between cat and dog trials [*t*(203) = 1.057, *p* = 0.212]. In real terms, this amounts to just a few more seconds required to complete the entire human RMET in comparison to the entire set of cats plus dogs (Supplementary Table 1).

### Reliability

Split half reliability (internal consistency) was 0.67 for the human RMET, 0.54 for cats and 0.59 for dogs; while Fleiss’ Kappa for inter-rater agreement was: human RMET = 0.44; cats = 0.39; dogs = 0.44 [fair agreement = 0.21–0.40; moderate agreement = 0.40–0.60; e.g., (51)].

To further test reliability of the newest stimuli i.e., the dog eyes test, extra data was collected for this task plus the human RMET in an additional sample of 228 undergraduate students (58 males and 170 females; mean age 19.87 years ± 1.04, median = 19.69, range = 18.26–24.07; age was not normally distributed, 95% CI: 19.59, 20.05). Accuracy for the dogs eyes stimuli was 72.45% (SE = 0.93%), and for the human RMET was 72.86% (*SE* = 0.76%), with no significant difference for accuracy [paired *t*(227) = 0.450, *p* = 0.650].

### Predictors of performance on the eyes tests

Correlations were calculated for each eyes stimulus set and all other measures (TOWRE A and B, TIWRE, HADS anxiety and depression subscales, the four IRI subscales, the three TAS subscales, the four O-LIFE subscales, the two IDAQ subscales, and total scores for the MRM, SAS-r, AQ50, and ECS). Stepwise linear regression models were then run with the score for each eyes stimuli set as DV, and scores for any correlated variables (Table 1) as IVs.

**TABLE 1:** Correlations between eyes stimuli set scores and other measures.

Eyes stimulus set	Correlated measure	*r*	*p*
Human	Test of Word Reading Efficiency part A	0.189	0.007
	Test of Word Reading Efficiency part B	0.189	0.007
	Test of Irregular Word Reading Efficiency	0.189	0.007
	Interpersonal Reactivity Index fantasy subscale	0.223	0.001*
	Toronto Alexithymia Scale externally oriented thinking	-0.235	<0.001*
	Individual Differences in Anthropomorphism Questionnaire non-anthropomorphic attributions	-0.146	0.037
Cat	Mind Reading Motivation scale	0.239	<0.001*
	Toronto Alexithymia Scale externally oriented thinking	-0.220	0.002*
	Individual Differences in Anthropomorphism Questionnaire non-anthropomorphic attributions	0.162	0.020
Dog	Toronto Alexithymia Scale externally oriented thinking	-0.208	0.003
	Individual Differences in Anthropomorphism Questionnaire non-anthropomorphic attributions	0.193	0.006
	Mind Reading Motivation scale	0.209	0.003

KEY: *r* = Pearson’s correlation coefficient; *df* = 202.

The best models were identified based on highest *R*^2^-values with all predictors making a significant contribution to the model. Human RMET [*F*(3,200) = 9.736, *p* < 0.001, adj *R*^2^ = 0.114] scores were predicted by TAS EOT (β = −0.208, *t* = −3.097, *p* = 0.002), TOWRE-A (β = 0.196, *t* = 2.964, *p* = 0.003), and IRI fantasy scores (β = 0.187, *t* = 2.782, *p* = 0.006). There were two predictors in the best model for the cat eyes stimuli [*F*(2,201) = 8.648, *p* < 0.001, adj *R*^2^ = 0.070] which were TAS EOT (β = −0.158, *t* = −2.197, *p* = 0.029) and MRM scores (β = −0.186, *t* = 2.586, *p* = 0.010), and for the dog stimuli set [*F*(2,201) = 7.797, *p* < 0.001, adj *R*^2^ = 0.063], which were EOT (β = −0.188, *t* = 2.744, *p* = 0.007), and IDAQ non-anthropomorphic attribution (β = 0.171, *t* = 2.499, *p* = 0.013) scores.

**FIGURE 2:**
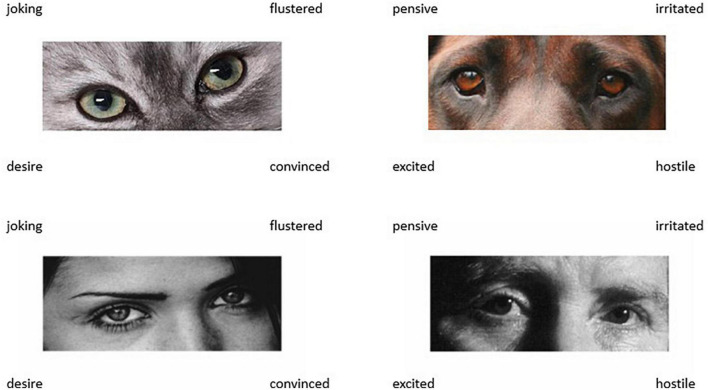
Example trials from the NARMET **(upper)** as compared to the RMET. Images were available without reuse restrictions. NARMET images adapted from https://pxhere.com/en/photo/979960, animals.com/animals/bavarian-mountain-hound/pictures/, and RMET images from https://www.autismresearchcentre.com/tests/eyes-test-adult/, licensed under CC0, CC BY-SA 3.0 and CC0 respectively.

Combining the 18 trial cats eyes test with the set of 18 dog eyes trials creates the Non-human Animal Reading the Mind in the Eyes Test (NARMET: Figure 2), which is the same length as the original 36 trial human RMET. Predictors of NARMET total scores were the predictors for cat and dog stimuli combined [*F*(3,200) = 9.474, *p* < 0.001, adj *R*^2^ = 0.111], i.e., TAS EOT (β = −0.181, *t* = 2.568, *p* = 0.011), MRM (β = −0.167, *t* = 2.305, *p* = 0.022), and IDAQ non-anthropomorphic attributions (β = 0.154, *t* = 2.235, *p* = 0.027).

### Additional characteristics of the new Non-human Animal RMET

The correct answers for the NARMET and the RMET (see Supplementary Table 2) were compared in terms of word length, frequency, valence, and concreteness using ratings from the English Lexicon Project (51). Ratings were available for all correct answers except fantasizing, which appears once in both the RMET and NARMET. Some other words were only available as verbs (e.g., insist versus insisting and regret versus regretful). Mean word length (RMET: 8.65; NARMET: 8.46), frequency (RMET: 7.51; NARMET: 7.46), and concreteness (RMET: 2.10; NARMET: 2.13) did not significantly differ between the RMET and NARMET. There was a slightly greater proportion of correct answers with a more obvious negative valence on the NARMET (RMET 4.89; NARMET: 4.30). Although the forced choice option combinations used in the RMET are also used in the NARMET, the number of unique correct answers after refinement is a little lower for the NARMET (RMET: 92%; NARMET: 69%). Finally, when considering other visual characteristics, there is also a difference in terms of the amount of stimuli depicting direct eye gaze (RMET: 66%; NARMET: 47%). Although these small differences are present they did not result in performance differences across cat, dog and human stimuli.

## Discussion

Building on the development of the cat eyes test (9), this study presents the new Non-Human Animal RMET. Together, the cat and dog stimuli comprise a 36 item test which can be used as a comparison measure alongside the original 36 item human RMET (1). The NARMET contains a similar range of complex mental states to the RMET such as suspicious, tentative, regretful, and defiant. Mental state judgments for NARMET stimuli are valid given that there was no significant differences in respondent accuracy compared to the standard RMET. This initial assessment of the NARMET indicates favorable psychometric properties in addition to demonstrating that the task is well matched to the RMET in terms of linguistic variables, plus overall difficulty.

Given that it was possible to create the NARMET, this may tell us something about the processes involved in these eyes tasks. Individuals appear to share a common interpretation of a fairly complex level of psychological state as reflected in the eyes of both humans and at least domesticated non-human animals such as cats and dogs. This ability seems unlikely to be a specialist skill, according to the degree of consensus seen across a few different samples during the development of the NARMET. It is still debated whether the RMET measures Theory of Mind, or emotion/mental state recognition, with difficulties posed by either interpretation. For example, Theory of Mind is often a more appropriate label when the cues are more abstract than visual (e.g., hearing a story, considering abstract mental states, like beliefs). On the other hand, visual cues may be more likely to prompt a recognition matching process. The issue with this latter interpretation is that we do not know whether the mental states that are being attributed to the eyes stimuli are indeed correct, and this is the case for both the RMET and NARMET stimuli, as correct responses are simply based on consensus. In the current study, response times were recorded, although most studies do not record this data. It took little more than 3 s of presentation time to per trial to elicit an accurate response, and this was consistent for both the human and non-human animal trials. This implies a fast and automatic underlying process, and perhaps reliance on a more instinctive mirroring type mechanism versus a more reasoned abstract perspective taking process, as the latter would likely take more time due to careful evaluation of each of the verbal labels in turn. Performance on the NARMET suggests that there are some fairly reliable visual cues (e.g., appearance of a low brow, gaze direction, and intensity) that individuals may use to draw conclusions about likely mental state, and that these cues may be more crude, and/or widely perceptible, than previously thought.

Although the range of mental states thought to be attributable to the cats and dogs was slightly less wide ranging than for humans, it was still quite extensive. This aligns with research showing that humans can attribute secondary emotions such as jealousy and guilt to dogs (25). While some studies have suggested that anthropomorphic judgments made by humans may be quite inaccurate [e.g., the guilty dog look: (52)], NARMET stimuli judgments were not related to mental state attributions on the anthropomorphism measure. Previous studies have shown that healthy participants activate many of the same brain areas (i.e., prefrontal and anterior temporal) when passively viewing humans, dogs, and primates (53) and when processing the expressions or biological motion of humans and animal faces including those of cats and dogs (54–57). However, there are potential differences in visual attention (58) and the neural correlates of face processing (59) when comparing human and non-human animals in children diagnosed with autistic spectrum disorders. Future fMRI studies may shed further light into any differences in the mental state attribution process across human and non-human eyes stimuli by using the NARMET alongside the RMET and the previously developed Age Eyes Test (9, 13, 14).

Mental state attribution in relation to human, cat and dog stimuli were all negatively associated with externally oriented thinking. When cats and dogs were combined into a single task, NARMET scores were predicted by mind reading motivation and non-anthropomorphic attributions, while human RMET performance was predicted by fantasy and word reading efficiency. Therefore, while NARMET performance may be more clearly associated with motivational factors, it is perhaps less susceptible to confounds related to abstraction, language or verbal skills, which can influence RMET performance (60). It may also avoid some of the cultural drawbacks [e.g., (61)] of the RMET. Further investigation is needed into its psychometric properties in additional samples, especially given the significant limitations such as the high majority of females, in addition to e.g., less than 2% of the sample showing any evidence of autistic tendencies. While some studies have found an effect of gender on RMET performance [e.g., (62–64)], others have not (9, 65, 66). Either way, future studies involving clinical or more diverse groups may identify specific predictors of performance on the NARMET, or on its two (cat and dog) subtests.

In conclusion, using the NARMET alongside the RMET (1), and the Age Eyes Test (9, 13, 14) could allow for the testing of double-dissociations based on making equivalent mental state judgments about different (human versus non-human animal) stimuli, versus different judgments (mental state versus physical state) about the same stimuli. It is anticipated that future studies employing this combination of tasks in developmental and clinical contexts could help to control for some performance confounds, offering enhanced assessment of social cognitive skills and unique insight into the processes involved in mental state attribution based on facial cues.

## Data availability statement

The raw data supporting the conclusions of this article will be made available by the authors, without undue reservation.

## Ethics statement

The studies involving human participants were reviewed and approved by the University of Birmingham Research Ethics Committee. The patients/participants provided their written informed consent to participate in this study.

## Author contributions

CE conceived and designed the study, prepared materials for the data collection, performed statistical analysis, interpreted the findings, and wrote the first and final draft of the manuscript.
