# Supplementary material for: The non-human animal reading the mind in the eyes test (NARMET): A new measure for the assessment of social cognition
Source: Front Psychiatry. 2023 Mar 20;14:1129252. doi: 10.3389/fpsyt.2023.1129252 (PMC10069677; doi:10.3389/fpsyt.2023.1129252)
Supplement: Supplementary file 1 [file Table_1.docx]

**Supplementary Table 1. Descriptive statistics for additional measures**

|  | MEAN | STANDARD ERROR | MEDIAN | RANGE |
| --- | --- | --- | --- | --- |
| TOWRE A | 90.43 | 0.65 | 91.5 | 57-104 |
| TOWRE B | 56.49 | 0.45 | 58 | 21-63 |
| TIWRE | 35.96 | 0.16 | 36 | 28-39 |
| HADS Anxiety | 7.14 | 0.28 | 7 | 0-20 |
| HADS Depression | 3.13 | 0.19 | 2 | 0-13 |
| IRI FS | 18.40 | 0.34 | 19 | 7-28 |
| IRI PT | 19.48 | 0.32 | 20 | 3-28 |
| IRI PD | 13.93 | 0.32 | 14 | 1-28 |
| IRI EC | 22.10 | 0.29 | 22 | 0-28 |
| TAS DIF | 15.25 | 0.41 | 15 | 7-34 |
| TAS DDF | 13.32 | 0.34 | 13 | 5-25 |
| TAS EOT | 17.21 | 0.28 | 17 | 9-28 |
| rSAS | 7.29 | 0.42 | 5 | 0-33 |
| AQ50 | 15.40 | 0.46 | 15 | 3-43 |
| ECS | 45.74 | 0.37 | 45 | 29-59 |
| IDAQ | 47.01 | 1.42 | 44 | 4-103 |
| IDAQ NA | 83.11 | 1.14 | 84 | 17-117 |
| MRM | 67.08 | 0.63 | 68 | 38-91 |
| O-LIFE UE | 2.93 | 0.18 | 3 | 0-11 |
| O-LIFE CD | 5.47 | 0.20 | 5 | 0-11 |
| O-LIFE IA | 1.47 | 0.12 | 1 | 0-8 |
| O-LIFE IN | 3.29 | 0.14 | 3 | 0-9 |

KEY= AQ50: Autism Spectrum Quotient; ECS: Emotional Contagion Scale; HADS: Hospital Anxiety and Depression Scale (anxiety or depression subscale); IDAQ: Individual Differences in Anthropomorphism Questionnaire (NA: non-anthropomorphising attributions subscale); IRI: Interpersonal Reactivity Index; MRM: Mind Reading Motivation scale; O-LIFE: Oxford-Liverpool Inventory of Feelings and Experiences (UE: unusual experiences; CD: cognitive disorganization; IA: inappropriate affect; IN: impulsive non-conformity); rSAS: Revised Social Anhedonia Scale; TAS: Toronto Alexithymia Scale (EOT: externally oriented thinking; DIF: difficulty identifying feelings; DDF: difficulty describing feelings); TIWRE: Test of Irregular Word Reading Efficiency; TOWRE: Test of Word Reading Efficiency (part A or B).

**Supplementary Table 2. The NARMET as compared to the RMET**

| Item number and correct answer | RMET | | | | Corresponding RMET item/ answer set | NARMET | | | | NARMET Correct Answer |
| --- | --- | --- | --- | --- | --- | --- | --- | --- | --- | --- |
|  | 1 | 2 | 3 | 4 |  | 1 | 2 | 3 | 4 |  |
| 1 playful | **51.5*** | 34.8 | 2.9 | 10.8 | 2 | 15.7 | **81.9** | 0.5 | 2.0 | upset |
| 2 upset | 12.7 | **79.9*** | 3.4 | 3.9 | 3 | 4.9 | 10.3 | **68.1** | 16.7 | desire |
| 3 desire | 3.9 | 5.9 | **77.5*** | 12.7 | 4 | 0.5 | **86.3** | 6.9 | 6.4 | insisting |
| 4 insisting | 2.9 | **76.0*** | 9.3 | 11.8 | 5 | 4.9 | 0.0 | **91.2** | 3.9 | worried |
| 5 worried | 6.9 | 8.8 | **83.3*** | 1.0 | 6 | 4.4 | **59.8** | 28.4 | 7.4 | fantasising |
| 6 fantasising | 2.5 | **72.1*** | 20.6 | 4.9 | 7 | 9.3 | 27.9 | **60.3** | 2.5 | uneasy |
| 7 uneasy | 3.9 | 27.5 | **59.8*** | 8.8 | 10 | **56.9** | 21.1 | 4.4 | 17.6 | cautious |
| 8 despondent | **72.5*** | 13.2 | 12.7 | 1.5 | 12 | 8.8 | 2.5 | **79.4** | 9.3 | sceptical |
| 9 preoccupied | 7.8 | 4.9 | 3.4 | **83.8*** | 13 | 12.7 | **57.4** | 16.2 | 13.7 | anticipating |
| 10 cautious | **52.5*** | 32.4 | 9.8 | 5.4 | 15 | **77.5** | 10.3 | 8.8 | 3.4 | contemplative |
| 11 regretful | 6.9 | 13.7 | **75.5*** | 3.9 | 19 | 15.2 | 15.2 | 10.8 | **58.8** | tentative |
| 12 sceptical | 9.8 | 2.9 | **83.8*** | 3.4 | 23 | 4.4 | 2.5 | **79.4** | 13.7 | defiant |
| 13 anticipating | 11.8 | **67.2*** | 3.4 | 17.6 | 26 | 8.8 | 2.0 | **84.3** | 4.9 | hostile |
| 14 accusing | 13.2 | 16.2 | 3.4 | **67.2*** | 27 | 3.9 | **43.6** | 33.3 | 19.1 | cautious |
| 15 contemplative | **84.3*** | 3.9 | 7.8 | 3.9 | 29 | 13.7 | 4.4 | **68.6** | 13.2 | irritated |
| 16 thoughtful | 4.4 | **77.5*** | 8.3 | 9.8 | 32 | **83.3** | 4.9 | 3.9 | 7.8 | serious |
| 17 doubtful | **70.6*** | 9.3 | 15.7 | 4.4 | 35 | 27.0 | **63.7** | 6.4 | 2.9 | nervous |
| 18 decisive | **66.2*** | 11.3 | 5.9 | 16.7 | 36 | 4.4 | 2.5 | **89.7** | 3.4 | suspicious |
| 19 tentative | 16.2 | 14.7 | 12.7 | **56.4** | 1 | **57.8** | 30.9 | 2.0 | 9.3 | playful |
| 20 friendly | 15.2 | **74.5*** | 9.3 | 1.0 | 2 | 13.7 | **82.8** | 1.5 | 2.0 | upset |
| 21 fantasising | 2.9 | **94.1*** | 2.0 | 1.0 | 5 | 2.9 | 2.9 | **83.3** | 10.8 | worried |
| 22 preoccupied | **74*** | 2.0 | 3.4 | 20.6 | 9 | 8.8 | 3.4 | 1.0 | **86.8** | preoccupied |
| 23 defiant | 16.2 | 8.3 | 34.8* | **40.7** | 11 | 20.6 | 2.9 | **74.0** | 2.5 | regretful |
| 24 pensive | **78.9*** | 8.8 | 4.9 | 7.4 | 13 | 8.8 | **57.8** | 19.1 | 14.2 | anticipating |
| 25 interested | 3.4 | 11.3 | 10.3 | **75*** | 15 | **83.3** | 8.8 | 3.4 | 4.4 | contemplative |
| 26 hostile | 8.3 | 5.9 | **70.1*** | 15.7 | 17 | **69.1** | 18.6 | 6.4 | 5.9 | doubtful |
| 27 cautious | 2.5 | **76*** | 10.3 | 11.3 | 19 | 2.9 | 32.8 | 2.5 | **61.8** | tentative |
| 28 interested | **72.1*** | 2.0 | 21.1 | 4.9 | 20 | 3.9 | **62.3** | 29.4 | 4.4 | friendly |
| 29 reflective | 14.7 | 2.0 | 15.7 | **67.6*** | 23 | 6.4 | 0.5 | **73.5** | 19.6 | defiant |
| 30 flirtatious | 1.0 | **93.6*** | 2.5 | 2.9 | 24 | **69.6** | 17.2 | 3.9 | 9.3 | pensive |
| 31 confident | 4.4 | **80.9*** | 4.9 | 9.8 | 26 | 7.4 | 0.5 | **86.8** | 5.4 | hostile |
| 32 serious | **83.8*** | 3.9 | 5.4 | 6.9 | 27 | 6.4 | **80.4** | 2.0 | 11.3 | cautious |
| 33 concerned | 2.5 | 16.7 | 10.3 | **70.6*** | 29 | 11.8 | 3.9 | 23.5 | **60.8** | reflective |
| 34 distrustful | 9.3 | 11.3 | **73.5*** | 5.9 | 30 | 13.2 | 12.3 | 12.3 | **62.3** | disappointed |
| 35 nervous | 10.8 | **45.6*** | 19.6 | 24.0 | 35 | 25.5 | **67.2** | 2.5 | 4.9 | nervous |
| 36 suspicious | 1.0 | 2.9 | **86.8*** | 9.3 | 36 | 1.5 | 4.9 | **83.8** | 9.8 | suspicious |

KEY: Showing correct answer* for RMET and highest consensus in bold. Non-human Animal RMET (NARMET) answers matched the correct answer for the original corresponding RMET item except for irritated and disappointed trials. The top 18 NARMET rows (shaded) are cat stimuli, the following 18 items are dogs.
